# Climate‐mediated population dynamics of a migratory songbird differ between the trailing edge and range core

**DOI:** 10.1002/ecm.1559

**Published:** 2023-01-04

**Authors:** William B. Lewis, Robert J. Cooper, Richard B. Chandler, Ryan W. Chitwood, Mason H. Cline, Michael T. Hallworth, Joanna L. Hatt, Jeff Hepinstall‐Cymerman, Sara A. Kaiser, Nicholas L. Rodenhouse, T. Scott Sillett, Kirk W. Stodola, Michael S. Webster, Richard T. Holmes

**Affiliations:** ^1^ Warnell School of Forestry and Natural Resources University of Georgia Athens Georgia USA; ^2^ Independent Researcher; ^3^ New Mexico Department of Game and Fish Santa Fe New Mexico USA; ^4^ Vermont Center for Ecostudies Norwich Vermont USA; ^5^ Cornell Laboratory of Ornithology Cornell University Ithaca New York USA; ^6^ Department of Biological Sciences Wellesley College Wellesley Massachusetts USA; ^7^ Smithsonian Migratory Bird Center National Zoological Park Washington District of Columbia USA; ^8^ Illinois Natural History Survey University of Illinois Champaign Illinois USA; ^9^ Cornell Laboratory of Ornithology and Department of Neurobiology and Behavior Cornell University Ithaca New York USA; ^10^ Department of Biological Sciences Dartmouth College Hanover New Hampshire USA

**Keywords:** apparent survival, black‐throated blue warbler, climate change, migratory songbird, population dynamics, range contractions, range core, recruitment, trailing edge

## Abstract

Understanding the demographic drivers of range contractions is important for predicting species' responses to climate change; however, few studies have examined the effects of climate change on survival and recruitment across species' ranges. We show that climate change can drive trailing edge range contractions through the effects on apparent survival, and potentially recruitment, in a migratory songbird. We assessed the demographic drivers of trailing edge range contractions using a long‐term demography dataset for the black‐throated blue warbler (*Setophaga caerulescens*) collected across elevational climate gradients at the trailing edge and core of the breeding range. We used a Bayesian hierarchical model to estimate the effect of climate change on apparent survival and recruitment and to forecast population viability at study plots through 2040. The trailing edge population at the low‐elevation plot became locally extinct by 2017. The local population at the mid‐elevation plot at the trailing edge gradually declined and is predicted to become extirpated by 2040. Population declines were associated with warming temperatures at the mid‐elevation plot, although results were more equivocal at the low‐elevation plot where we had fewer years of data. Population density was stable or increasing at the range core, although warming temperatures are predicted to cause population declines by 2040 at the low‐elevation plot. This result suggests that even populations within the geographic core of the range are vulnerable to climate change. The demographic drivers of local population declines varied between study plots, but warming temperatures were frequently associated with declining rates of population growth and apparent survival. Declining apparent survival in our study system is likely to be associated with increased adult emigration away from poor‐quality habitats. Our results suggest that demographic responses to warming temperatures are complex and dependent on local conditions and geographic range position, but spatial variation in population declines is consistent with the climate‐mediated range shift hypothesis. Local populations of black‐throated blue warblers near the warm‐edge range boundary at low latitudes and low elevations are likely to be the most vulnerable to climate change, potentially leading to local extirpation and range contractions.

## INTRODUCTION

Anthropogenic climate change has caused range shifts across a wide range of taxonomic groups (Chen et al., 2011; Hickling et al., 2006; Parmesan & Yohe, 2003; Platts et al., 2019; Root et al., 2003; Vitasse et al., 2021). The long‐term consequences of such widespread range shifts are uncertain, although predicted effects include loss of biodiversity and degradation of ecosystem function (Bellard et al., 2012; Pecl et al., 2017). Individual species show a large degree of heterogeneity in the direction and magnitude of range shifts (Tingley et al., 2012; Vanderwal et al., 2013), but most shifts have been toward higher latitudes and elevations (Chen et al., 2011; Freeman et al., 2018; Hastings et al., 2020; Hickling et al., 2006; Parmesan et al., 1999; Vitasse et al., 2021). Among species' ranges that have shifted toward cooler areas, some have expanded at the leading edge (increasing range size), some have contracted at the trailing edge (decreasing range size), and some have done both (maintaining similar range size) (Coristine & Kerr, 2015; Freeman et al., 2018; Parmesan et al., 1999; Rushing et al., 2020). Gaining a better understanding of the mechanisms driving range shifts will help elucidate the drivers of these heterogeneous responses and is critically important for predicting and managing future range shifts under climate change.

Range shifts are population‐level processes that result from spatial and temporal variation in demographic rates (Gaston, 2009; Schurr et al., 2012; Sexton et al., 2009). Peripheral populations are nearest to the biotic and abiotic conditions which interact to set range limits, and so are nearer to their ecological tolerances compared to populations in the range core (Brown, 1984; Holt et al., 2005; Lee‐Yaw et al., 2016; Sirén & Morelli, 2020). Climate is an important determinant of range boundaries at both the cool and warm edges of the range, either directly (e.g., via thermal stress) or indirectly through biotic interactions (e.g., competition, food) (Cahill et al., 2014; Coristine & Kerr, 2015; Cunningham et al., 2016; Mac Arthur, 1972; Wiens, 2011). Climate change causes range shifts by altering the geographic distribution of the factors setting range limits (Cahill et al., 2014). This leads to spatial differences in demographic rates between the periphery and range core that can drive local colonization and range expansion at the leading edge and local extinction and range contraction at the trailing edge (Gaston, 2009; Schurr et al., 2012).

Understanding the demographic drivers of range shifts is especially important at the trailing edge, where climate change often has the strongest negative effects (Anderson et al., 2009; Jiguet et al., 2010). Trailing edge populations that cannot disperse or adapt to climate‐mediated changes in biotic interactions and abiotic conditions are likely to decline via reduced vital rates or increased emigration (Cahill et al., 2013; Chown et al., 2010; Coristine & Kerr, 2015). Loss of trailing edge populations may decrease regional biodiversity (Hampe & Petit, 2005; Merker & Chandler, 2020) and genetic diversity because these populations are frequently genetically distinct from populations in other parts of the range (Ferrari et al., 2018; Hampe & Petit, 2005; Parisod & Joost, 2010). Incorporating demography into predictive models should allow for insights into the mechanisms of population declines and increase the forecasting ability for trailing edge range contractions (Normand et al., 2014; Schurr et al., 2012; Urban et al., 2016), but few studies of animal populations have been designed to examine spatial and temporal variation in demographic rates at the trailing edge.

Two factors have impeded the study of the demographic drivers of trailing edge range contractions. First, a change in a demographic rate may have little effect on range dynamics if declines in one vital rate are offset by density‐dependent increases in others (Aikens & Roach, 2014; Doak & Morris, 2010; Villellas et al., 2015). Studies investigating the demographic drivers of range shifts must incorporate relevant demographic rates and establish a direct link between climate change, demographic rates, and spatial variation in population dynamics (Mclean et al., 2016; Pironon et al., 2018). Second, demographic studies are often restricted to small spatial areas or short time periods (Clutton‐Brock & Sheldon, 2010); however, the broad temporal and spatial scale of range shifts necessitates the collection of demographic data from across the geographic range (e.g., trailing edge and range core) and over long enough time periods to measure responses to climate change (Ehrlén & Morris, 2015; Oldfather & Ackerly, 2019; Purves, 2009). Currently, studies documenting trailing edge demographic responses to climate change have mostly focused on plants (e.g., Doak & Morris, 2010; Oldfather & Ackerly, 2019; Sheth & Angert, 2018). Demographic responses to climate change may differ between plants and mobile animals that can respond to climate change through movement (Charmantier et al., 2008; Refsnider & Janzen, 2012; Rushing et al., 2015).

Here, we model the demographic drivers of range contractions in a migratory bird species. We test two hypotheses using long‐term demographic data collected across a range of elevations from study populations in two regions of the geographic range (hereafter, range positions): the trailing edge and the range core. First, we hypothesize that local demographic rates are sensitive to climate. Within each range position, we expect local demographic rates to be correlated with variations in temperature and precipitation. Second, we hypothesize that populations at the trailing edge are nearer to their ecological tolerance than populations at the range core. We predict that trailing edge populations are more sensitive to changes in climate, leading to local extirpations and trailing edge range contractions. Specifically, if trailing edge populations are more sensitive to climate, then changes in temperature and precipitation at the trailing edge should lead to declining recruitment, apparent survival, and density compared with populations in the range core. Furthermore, the negative effects of climate should be strongest, and first observed, at the warmest and driest habitats at low elevations within the trailing edge. We tested the two hypotheses using an 18‐year dataset on the breeding demography of black‐throated blue warblers (*Setophaga caerulescens*) collected across a range of elevations at both range positions.

## METHODS

### Study species

Black‐throated blue warblers are small (9–10 g) migratory songbirds that have been used as a model species for studying demographics and population regulation (reviewed in Holmes, 2007, 2011). The breeding range consists of temperate deciduous and mixed forests in the northern USA and southern Canada, with trailing edge populations in the southern Appalachian Mountains (Holmes et al., 2017). Adult birds are sexually dimorphic, territorial, and socially monogamous, although <20% of males may be bigamous (Holmes et al., 1992). Little is known about juvenile dispersal, but studies from other species of songbirds indicate that natal dispersal distances are usually <100 km (Paradis et al., 1998; Tittler et al., 2009). In addition, the recruitment of yearling black‐throated blue warblers at the range core is significantly and positively correlated with per‐capita fecundity in the previous breeding season (Holmes et al., 1992; Sillett et al., 2000; Sillett & Holmes, 2005). We therefore assumed that most yearlings recruited to the study plots were hatched in the same general area (i.e., within 100 km). Populations have been declining at the trailing edge of the range while remaining stable in the range core (Sauer et al., 2017); however, the causes of these declines are not well understood.

### Study sites

**FIGURE 1:**
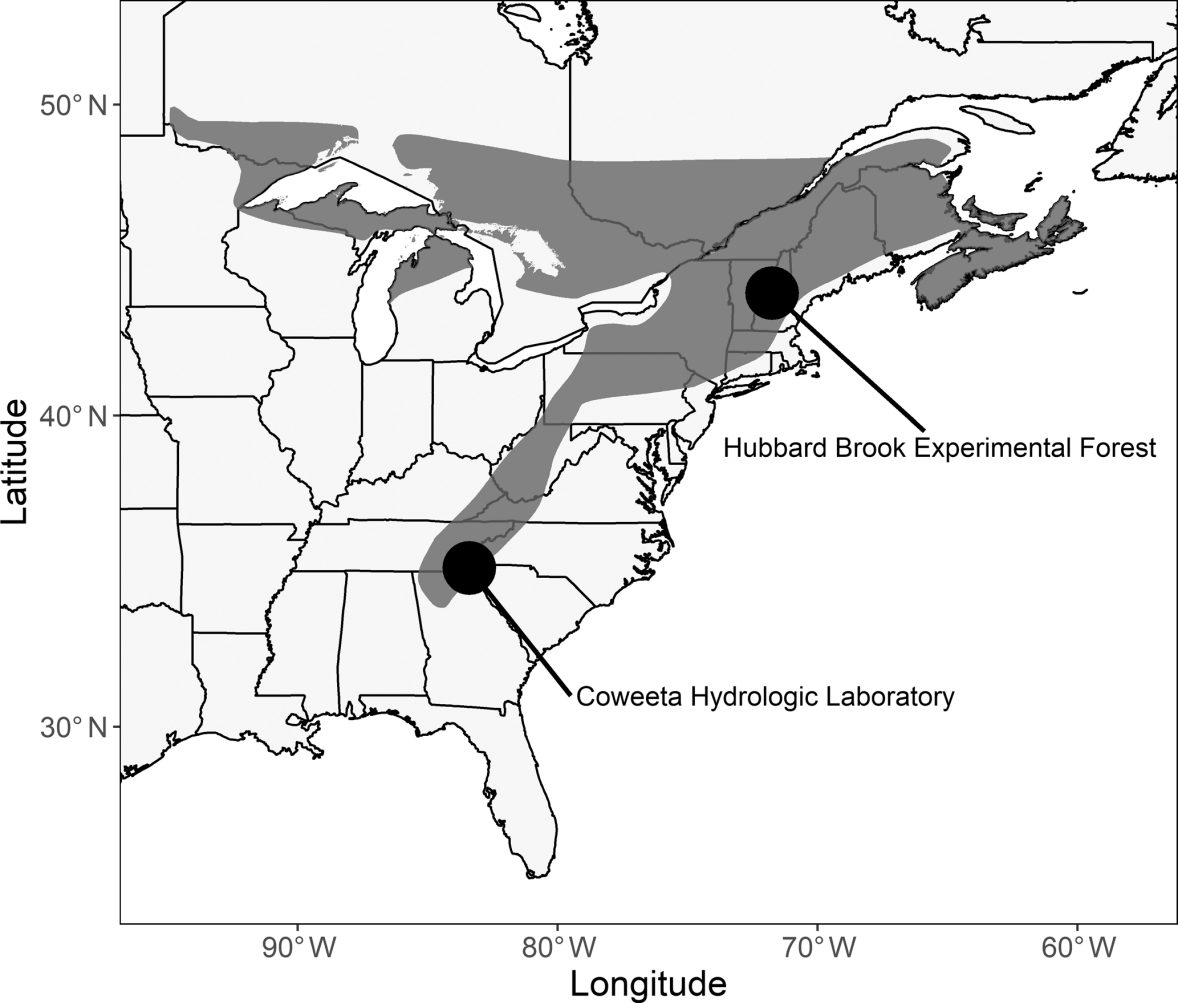
Geographic location of demographic study plots within the breeding range (shaded) of black‐throated blue warblers (*Setophaga caerulescens*). The range map was provided by the NatureServe and IUCN (International Union for Conservation of Nature) (2022). The Coweeta Hydrologic Laboratory is located at the trailing edge of the range in the southern Appalachian Mountains in North Carolina while the Hubbard Brook Experimental Forest is located within the core of the range in New Hampshire.

Demographic data were collected from 2002 to 2019 at two range positions (Figure 1): the southern, trailing edge of the range near the Coweeta Hydrologic Laboratory in the Nantahala National Forest in the Appalachian Mountains of North Carolina, USA (35.1° N, 83.4° W) and the core of the range at the Hubbard Brook Experimental Forest in New Hampshire, USA (43°56′ N, 71°45′ W). Both range positions have experienced long‐term increases in temperature over the past several decades (Ford et al., 2011; Townsend et al., 2016).

Study sites are composed of contiguous northern hardwood forest at the range core and contiguous cove hardwood and northern hardwood forest at the trailing edge (Bormann & Likens, 1979; Day & Monk, 1974). The range core is characterized by an overstory of sugar maple (*Acer saccharum*), American beech (*Fagus grandifolia*), and yellow birch (*Betula alleghaniensis*), with an understory of hobblebush (*Viburnum lantanoides*) and saplings of striped maple (*Acer pensylvanicum*) and the major overstory species (van Doorn et al., 2011). The community of dominant canopy species at the trailing edge is more diverse than at the range core. The canopy at the trailing edge is composed mainly of oak (*Quercus* spp.), hickory (*Carya* spp.), and red maple (*Acer rubrum*), although all major tree species at the range core are also present. Understory at the trailing edge is predominantly rhododendron (*Rhododendron maximum*) and mountain laurel (*Kalmia latifolia*) (Day & Monk, 1974). Within both range positions, three large plots were established across an elevation gradient roughly spanning the breeding elevation range of black‐throated blue warblers (Table 1). We refer to the collection of black‐throated blue warblers breeding at each study plot as a “local population.” The boundaries of many study plots changed slightly over the study period; however, we only consider data from the long‐term portions of the study plots that were surveyed every year. The one exception to this was the mid‐elevation plot at the trailing edge (see below). Many variables that affect black‐throated blue warbler demography, such as the distribution of food and nesting vegetation, are correlated with the elevational gradient at both range positions (Cline et al., 2013; R. Cooper, unpublished data; Holmes, 2011; Kaiser et al., 2015; Stodola et al., 2013); however, these elevational patterns likely arise, at least partially, in response to the elevational climate gradient. This sampling design enabled the collection of demographic data near the low‐elevation range limit at both range positions, but birds breeding at the low‐elevation plot at the trailing edge were near both the elevational and latitudinal edge of the breeding range.

**TABLE 1:** Description of black‐throated blue warbler (*Setophaga caerulescens*) census/capture–recapture plots across an elevation gradient near the trailing edge (North Carolina) and core (New Hampshire) of the species' breeding range.

Range position	Elev. class	Avg. elev.	Area	*N*	Sampling period	Early‐breed. temp.	Annual precip.
Trailing	Low	1050	23	15	2002–2008, 2017–2018	17.04 (0.91)	2190 (454)
	Mid	1200	18	61	2002–2019	16.14 (0.91)	2283 (474)
	High	1350	15	102	2003–2019	15.24 (0.92)	2376 (495)
Core	Low	300	98	161	2002–2016	16.19 (1.05)	1418 (181)
	Mid	550	125	428	2002–2016	15.29 (1.08)	1491 (192)
	High	800	56	209	2002–2016	14.39 (1.15)	1581 (209)

*Note*: Average elevation in meters above sea level (asl) (Avg. elev.), relative elevation class within each range position (Elev. class), and area of the study plots in hectares (area) are shown, as well as the number of marked females used in the analysis (*N*) and the time period over which each plot was sampled (Sampling period). Annual precipitation in millimeters (Annual precip.) and mean of average daily temperatures in °C during the early‐breeding season (Early‐breed. temp) are shown for each study plot, averaged across the years of the study (2002–2019). The early‐breeding season is defined as the average lay date to average fledge date of first broods at each range position. Climate variables are shown as mean (SD). Climate variables shown in the table are values predicted to the elevation of the study plots.

The mid‐elevation plot at the trailing edge was expanded by 11 ha in 2006. The original 18 ha section consisted largely of poor‐quality habitat; this section was dry with a predominantly open understory and scattered clumps of rhododendron, the preferred nesting substrate for black‐throated blue warblers at the trailing edge. The expanded section was predominantly higher quality habitat—wetter with the abundant cover of the preferred nesting substrate. Unless otherwise indicated, references to the mid‐elevation plot at the trailing edge refer to the original, largely poor‐quality section of the study plot.

### Field methods

Study plots were surveyed at least every other day during the breeding season each year to locate black‐throated blue warblers and their nests. The coordinates of all encountered individuals were recorded either using handheld GPS or on gridded plot maps that were subsequently digitized. Territory boundaries were delineated using coordinates from the encounter locations; territories were visited, on average, every other day. Individuals were captured using mist nets and marked with a USGS aluminum leg band and a unique combination of three colored leg bands to allow for individual identification at a distance without the need for recapture. Age and capture location data were recorded for each captured individual. Black‐throated blue warblers can be classified into two age classes based on plumage characteristics (Pyle, 1997): second‐year individuals (first‐time breeders, hereafter SY) or after‐second‐year individuals (at least second breeding year, hereafter ASY). The age class of some females could not be determined because they possessed intermediate plumage characteristics; these individuals were classified into a nonspecific age class (first‐time breeder or older, hereafter AHY). A few females were not captured each year; however, unmarked females could be individually identified within a year based on the location of their territories. Individuals were excluded from the analysis if more than half of their territories were outside the plot boundaries. These survey methods resulted in two types of data: individual encounter histories of marked individuals and yearly counts of unmarked individuals.

### Climate data

Climate data were collected from a series of USDA Forest Service climate stations situated across a range of elevations near study sites at each range position (Miniat et al., 2015, 2017; USDA Forest Service Northern Research Station, 2020a, 2020b). We only included a climate station in the analysis if the elevation of the climate station was within 175 m of the elevation of the study plots at each range position (Table 1; Appendix S1: Table S1), leaving nine stations at the trailing edge (three temperature, six precipitation) and 16 stations at the range core (six temperature, 10 precipitation).

**FIGURE 2:**
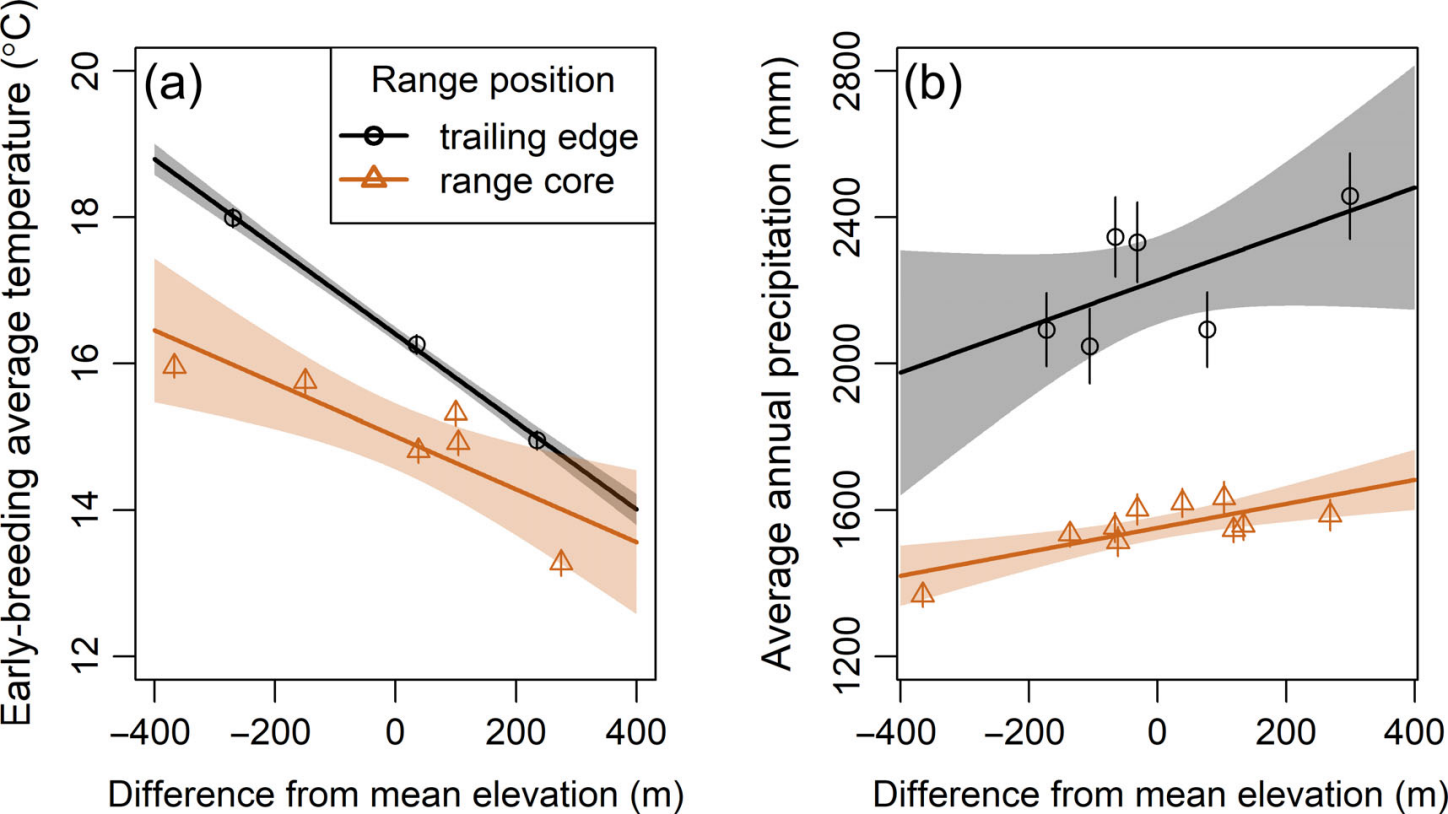
Mean of average daily temperature during the early‐breeding period (a) and mean annual precipitation (b) from 2002 to 2019 in relation to elevation at the trailing edge (North Carolina) and core (New Hampshire) of the breeding range of black‐throated blue warblers (*Setophaga caerulescens*). The early‐breeding season is the average lay date to average fledge date of first broods of black‐throated blue warblers, defined as day of year 127–159 (trailing edge) and 143–175 (range core). Trend lines and 95% CIs (shaded) from linear models relating elevation to climate variables at each range position are shown. The elevation breeding range of black‐throated blue warblers varied between range positions. To facilitate comparison across range positions, elevation is shown as the difference from the mean elevation of temperature (trailing edge mean: 1154 m; range core mean: 628 m) and precipitation (trailing edge: 1067 m; range core: 627 m) stations at each range position. Climate data were provided by long‐term climate stations run by the USDA Forest Service. See *Climate data* for more details.

**FIGURE 3:**
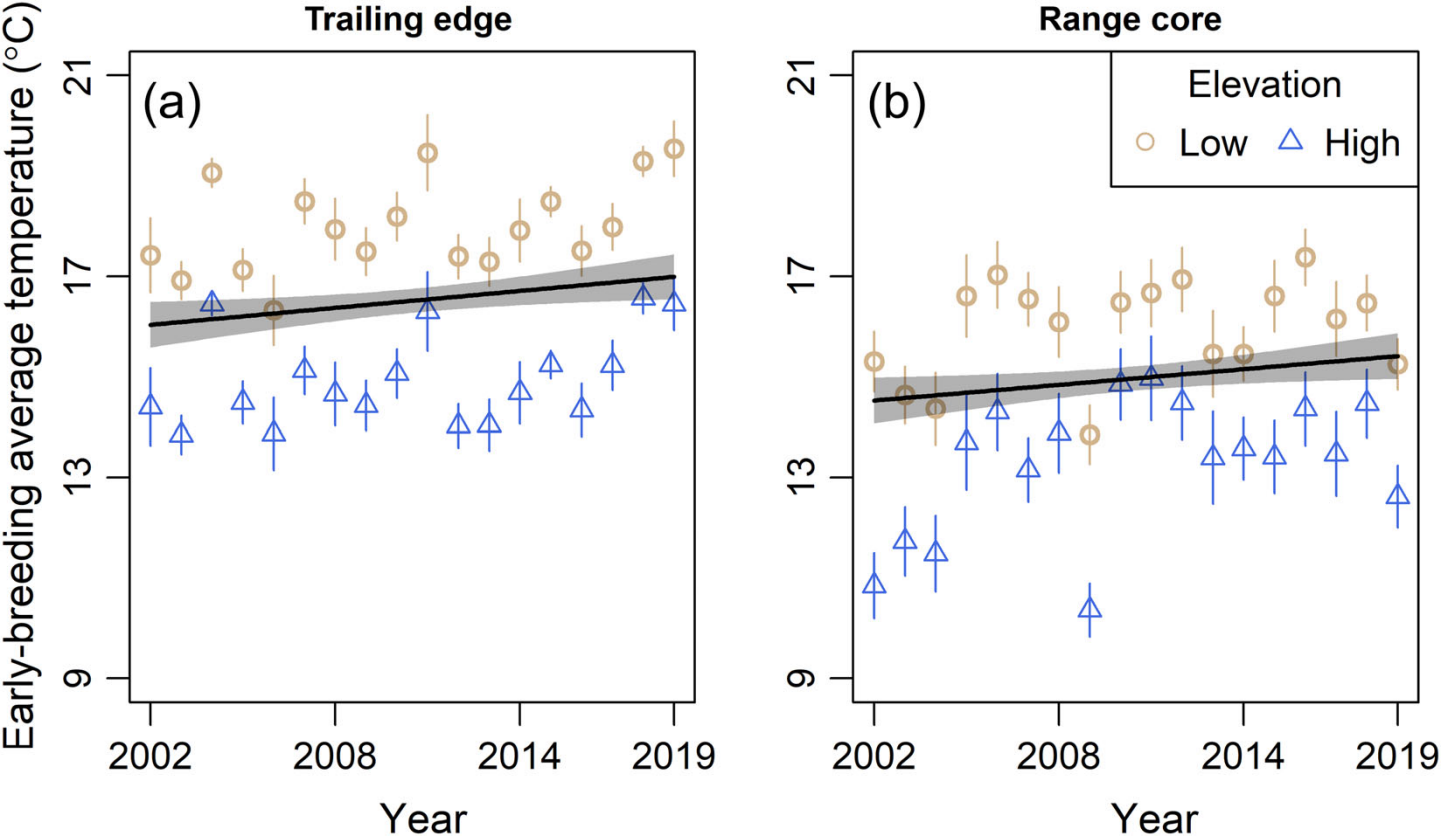
Change in the mean of average daily temperature during the early‐breeding period (average lay date to average fledge date of first broods of black‐throated blue warblers [*Setophaga caerulescens*]) from 2002 to 2019 at the trailing edge of the breeding range in North Carolina (a) and core of the breeding range in New Hampshire (b). Climate data were provided by long‐term climate stations run by the USDA Forest Service. Trend lines and 95% CIs (shaded) from linear models relating year to climate variables at each range position, accounting for elevation, are shown. Trend lines were predicted at the mean elevation of temperature stations at each range position (trailing edge: 1154 m; range core: 628 m). Mean and SE bars are shown for daily average temperature during the early‐breeding period recorded from temperature stations at each range position. For ease of visualization, temperature recordings are only shown for the lowest‐ and highest‐elevation temperature stations used in the analysis at each range position. See *Climate data* for more details.

**FIGURE 4:**
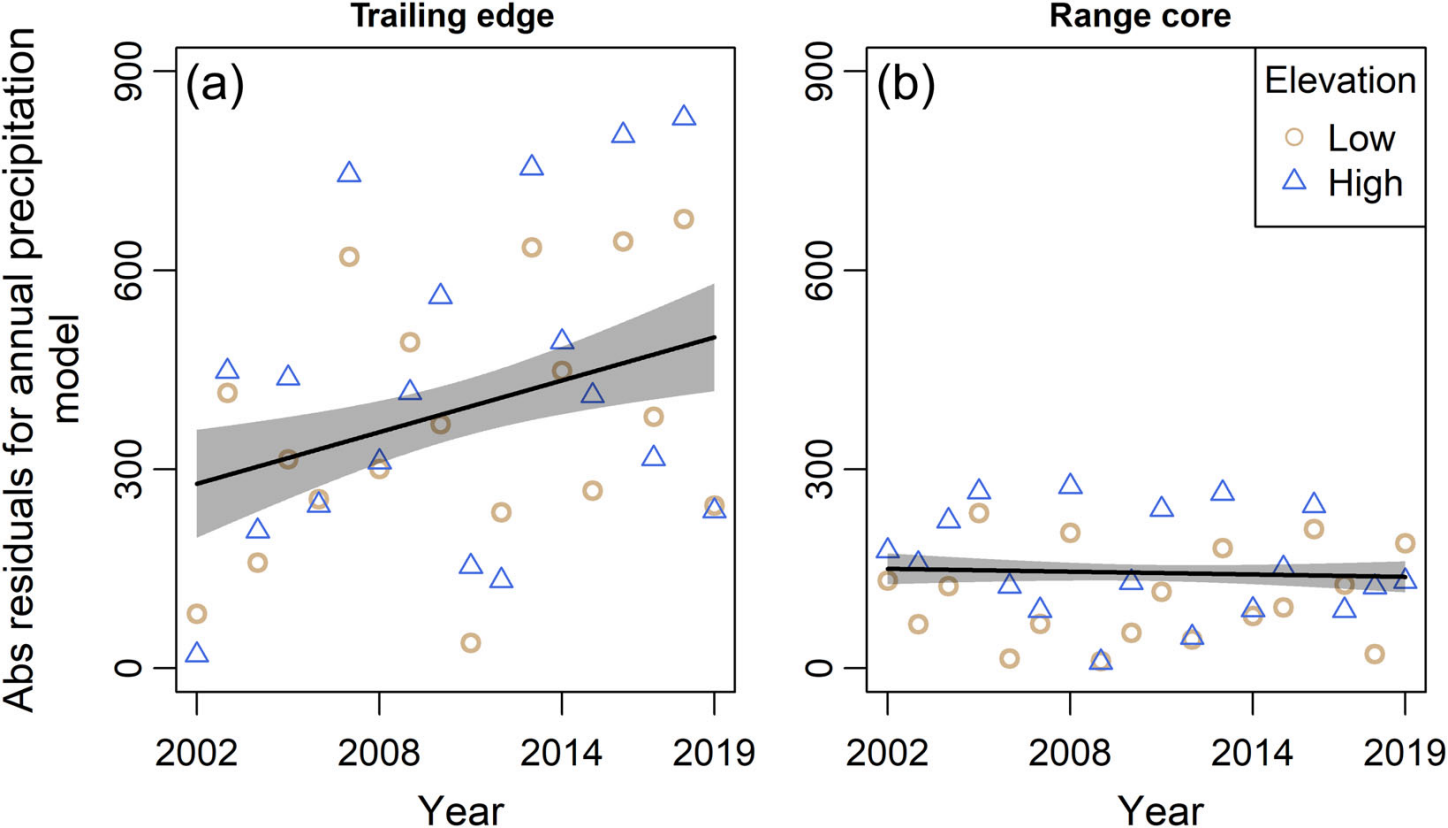
Variation in annual precipitation 2002–2019 at the trailing edge (North Carolina, a) and range core (New Hampshire, b) of the breeding range of black‐throated blue warblers (*Setophaga caerulescens*). Figures show absolute values of residuals from a linear model allowing for, at each range position, annual precipitation to vary by year (fixed effect) and elevation (trend effect). Absolute values of residuals are shown, as these were used as the dependent variable in an analysis to test if precipitation has become more variable over the study period. For ease of visualization, values are only shown for the lowest‐ and highest‐elevation precipitation stations used in the analysis at each range position. Trend line and 95% CIs (shaded) from a linear regression relating year to the absolute value of residuals, accounting for elevation, are shown. Trend lines were predicted at the mean elevation of precipitation stations at each range position (trailing edge: 1067 m; range core: 627 m). See *Climate data* for more details.

We incorporated two climate variables in the analysis: average daily temperatures during the early‐breeding period (hereafter, early‐breeding temperature) and annual precipitation. We calculated early‐breeding temperature as the mean of the average daily temperatures between the average first laying date (day of year 127 [trailing] and 143 [core]) and the average fledge date (day of year 159 [trailing] and 175 [core]) of first nesting attempts at each range position. Annual precipitation roughly corresponds to wet vs. dry years (less or more than 2100–2200 mm/year [trailing edge] and 1700 mm/year [range core], depending on elevation). These two variables are not the only possible climate metrics that may influence warbler breeding dynamics; however, the exact windows during which temperature and precipitation may affect warbler populations, and how these windows may differ between range positions, are somewhat unclear. We chose to focus our analyses on these two climate variables based on our a priori knowledge of the study system and which climate variables could be impactful on black‐throated blue warbler population dynamics. We included early‐breeding temperature in the analysis because this period of the annual cycle is the time of maximal breeding activity. Furthermore, early‐breeding temperatures were generally correlated (*r* > 0.6) with other temperature‐based variables during the breeding season (such as minimum daily temperature) for at least one range position. Rates of nestling starvation are higher in wetter years at the range core (Rodenhouse & Holmes, 1992), but the main food source of black‐throated blue warblers, caterpillars (order Lepidoptera), are more abundant in wetter years at the trailing edge (R. Cooper, unpublished data). The critical time period of the annual cycle during which precipitation may affect annual variation in demographic rates and population dynamics of black‐throated blue warblers is uncertain, especially at the trailing edge. Early‐breeding temperatures and annual precipitation were both higher at the trailing edge (Figure 2). We used linear regression models to assess long‐term changes in climate at each range position, accounting for elevation by including it as a covariate in models. Our measure of early‐breeding temperature is a mean value and so is associated with some uncertainty. We accounted for this uncertainty by calculating the standard deviation (SD) of the early‐breeding temperatures at each climate station, and weighting the mean estimates of early‐breeding temperature by the reciprocal of the SD in all regression models. Early‐breeding temperatures increased by 0.07°C/year (95% confidence interval [CI]: 0.03–0.11) at the trailing edge and 0.05°C/year (95% CI: 0.01–0.10) at the range core over the study period (Figure 3). Annual precipitation became more variable over the study period at the trailing edge but not at the range core (linear regression of residuals from a model specifying a time‐trend effect on annual precipitation; trailing edge: *t* = 3.12, *p* = 0.002; range core: *t* = −0.60, *p* = 0.55; Figure 4).

Climate stations were located near the study plots at the range core but not at the trailing edge; however, climate is broadly correlated with elevation at each range position (Figure 2). For each climate variable at each range position, we used the data from the climate stations to fit a linear model between elevation and the climate variable. This model was then used to predict the mean $\left(\mu_{t}^{\left(w\right)}\right)$ and standard error (SE) $\left(\sigma_{t}^{\left(w\right)}\right)$ of climate conditions at the elevation of the study plots each year. We specified a trend effect of elevation and a separate intercept for each year in the linear models.

### Statistical models of population dynamics

We used a Bayesian hierarchical model to draw inferences on black‐throated blue warbler population dynamics. The model structure was based on the Jolly–Seber model (Jolly, 1965; Kéry & Schaub, 2011; Seber, 1965), but modified to integrate both individual capture histories from marked individuals and counts of unmarked individuals. Models were female‐specific and estimated processes of population growth, recruitment, age‐specific apparent survival, capture probability, and temporary emigration (see below). Models were run separately for each study plot, ensuring that all parameters were estimated independently for each plot.

Female abundance each year was represented by $N_{t}$. The yearly abundance of the local population was a state variable that was not directly observed due to imperfect detection and temporary emigration, so we adopted a data augmentation approach (Royle et al., 2007). Data augmentation sets an upper limit (M) on the number of individuals that could have entered the local population over the entire study period; we set M at 600 to ensure that it was much larger than the actual number of individuals entering the local population. Yearly abundance was then given by $N_{t}=\sum_{i=1}^{M}z_{i,t}$, where $z_{i,t}$ was a binary state indicating if female i was alive in time t. Changes in abundance between t and t+1 were modeled based on recruitment rate (γ) and age‐specific apparent survival ($\phi_{j}$). Models were run three times at each study plot; for each model we specified a trend effect of either time, early‐breeding temperature, or annual precipitation on demographic rates. To account for uncertainty when predicting climate variables to the elevations of the study plots, climate variables were modeled as: $\mu_{t}^{\left(w\right)}∼\text{Norm}(w_{t},\sigma_{t}^{\left(w\right)})$.

### Initial abundance and recruitment

Abundance in the first year ($N_{1}$) was modeled as a function of the year that a female entered the population ($b_{i}$). Entry year for females was modeled as: $b_{i}∼\text{Categorical}\left(\pi_{1},\ldots ,\pi_{T+1}\right)$ where T was the number of years of data at the study plot. A female was alive in the first year according to $z_{i,1}=I\left(b_{i}=1\right)$. Female age ($a_{i,t}$) was given as 0 for SY and 1 for ASY. Age in the first year was modeled as $a_{i,1}∼\text{Bern}\left(\tau \right)$, where τ was an age‐ratio parameter representing the probability of a female being in the ASY age class. First‐year abundance was calculated as the sum of SY and ASY abundance in the first year, which were given by $n_{1,1}=\sum_{i=1}^{M}z_{i,1}\left(1-a_{i,1}\right)$ and $n_{2,1}=\sum_{i=1}^{M}z_{i,1}a_{i,1}$, respectively.

Entry of females into the population in subsequent years (t=2,…,T) was modeled based on the recruitment rate. The recruitment rate from year t to t+1 was defined by a logistic function specifying negative density dependence and either a temporal trend or a trend for one of the climate variables (denoted in equation as $w_{t}$):

(1) $$\gamma_{t}=\alpha /\left(1+\exp\left(-\left(\beta_{0}^{\left(\gamma \right)}+\beta_{1}^{\left(\gamma \right)}w_{t}-\beta_{2}^{\left(\gamma \right)}E{\left(D_{t}\right)}^{2}\right)\right)\right),$$

where α represented a bounding parameter and $E\left(D_{t}\right)$ was the expected density in t. The probability of entering a population in a year beyond the first was given by:

(2) $$\pi_{t}=\mathrm{Pr}\left(b_{i}=t\right)=\frac{E\left(n_{1,t}\right)}{M}=\frac{E\left(n_{1,t-1}\right)\gamma_{t-1}+E\left(n_{2,t-1}\right)\gamma_{t-1}}{M},$$

where $E\left(n_{1,t-1}\right)$ represented the expected abundance of SY females in time t−1 and $E\left(n_{2,t-1}\right)$ represented the expected abundance of ASY females in time t−1. The probability of an individual not entering the population over the course of the study was given by $\pi_{T+1}=1-\sum_{t=1}^{T}\pi_{t}$. Female age beyond the first year was fully specified by $b_{i}$, such that:

(3) $$a_{i,t}=\left\{\begin{array}{c} 0\;\left(\mathrm{SY}\right)\;\text{if}\;b_{i}=t\\ 1\;\left(\mathrm{ASY}\right)\;\text{otherwise} \end{array}\right..$$

This formulation classifies age as ASY when $b_{i}<t$; however, $a_{i,t}$ is multiplied by $z_{i,t}$ in the model so age class before $b_{i}$ does not influence the posterior distribution.

### Apparent survival

Apparent survival is the probability of surviving and not permanently emigrating from the local population. Age‐specific apparent survival from year t to t+1 was given by a logistic model with either a temporal trend or a trend for one of the two climate variables: $\text{logit}(\phi_{j,t})=\beta_{j,0}^{\left(\phi \right)}+\beta_{1}^{\left(\phi \right)}w_{t}$, where subscript j represented age class. Beyond the first year, females were alive in time t if they recruited in t or were alive in t−1 and returned in t:

(4) $$z_{i,t}∼\left\{\begin{array}{l} 0\;\text{if}\;b_{i}>t\\ 1\;\;\text{if}\;b_{i}=t\\ \text{Bern}\left(z_{i,t-1}\phi_{a_{\mathrm{it}},t-1}\right)\;\;\;\;\;\;\;\;\text{if}\;b_{i}<t \end{array}\right..$$

### Mark–resight process

Individuals were typically only captured once and marked, after which they were resighted without capture. Unmarked females were captured with probability $p_{t}^{\left(c\right)}$, and marked females were resighted on the study plots with probability $p^{\left(\eta \right)}$. Temporal variation in capture probability was modeled with a logit–linear function that included a temporal trend: $\text{logit}(p_{t}^{\left(c\right)})=\beta_{0}^{\left(c\right)}+\beta_{1}^{\left(c\right)}t$. Upon capture, unmarked females were classified into a specific age class (SY/ASY) rather than the nonspecific age class (AHY) with probability k. Age‐specific capture data were modeled as:

(5) $${\text{capture}}_{i,t}∼\left\{\begin{array}{l} \text{Bern}\left(z_{i,t}\times p_{t}^{\left(c\right)}\times k\right)\;\;\;\text{if classified into}\;\mathrm{SY}/\mathrm{ASY}\;\mathrm{age}\;\text{class}\\ \text{Bern}\left(z_{i,t}\times p_{t}^{\left(c\right)}\times \left(1-k\right)\right)\;\text{if classified into}\;\mathrm{AHY}\;\mathrm{age}\;\text{class}\; \end{array}\right..$$

Based on within‐season daily resighting data collected from 2011 to 2019 at the mid‐ and high‐elevation plots at the trailing edge, the average detection probability per visit for marked females was 0.61 (SD 0.27). This calculation excluded detections from females at the nest, as the detection probability for females attending a previously located nest is higher than when searching territories for females. Territories were visited 10–44 times over the course of a breeding season (median: 27); therefore, the probability of not detecting a female over the course of the entire season was likely extremely low (probability of not detecting a female over the entire season = (1–0.61)^10^, <0.001). Instead, $p^{\left(\eta \right)}$ largely represented the probability that a female was breeding on the study plot rather than having temporarily emigrated off the plot. Resight history data from marked females were modeled as: $y_{i,t}∼\text{Bern}\left(z_{i,t}\times p^{\left(\eta \right)}\right)$.

We also integrated yearly counts of unmarked females into estimates of $N_{t}$. The number of unmarked females in the population at time t was given by: $U_{t}=\sum_{i=1}^{M}z_{i,t}\left(1-m_{i,t}\right)$, where $m_{i,t}$ was a binary variable representing if female i was marked in time t. Yearly counts of unmarked females were then given by: $u_{t}∼\mathrm{Bin}\left(U_{t},p^{\left(\eta \right)}\right)$.

The low‐elevation plot at the trailing edge was not sampled after 2008 (see *Results*), but we incorporated follow‐up surveys performed in 2017 and 2018. In each of these years, this plot was intensively searched by 1–3 observers for 1 day during the middle of the breeding season. To account for detection bias in years when the plot was only surveyed once, we incorporated a detection probability term $\left(p^{\left(r\right)}\right)$ in the estimate of $y_{i,t}$ and $u_{t}$. Specifically, encounter history data were modeled as $y_{i,t}\;∼\;\text{Bern}\left(z_{i,t}\times p^{\left(\eta \right)}\times p^{\left(r\right)}\right)$ and counts of unmarked females were modeled as $u_{t}\;∼\;\mathrm{Bin}\left(U_{t},p^{\left(\eta \right)}\times p^{\left(r\right)}\right)$. An informative prior was placed on $p^{\left(r\right)}$ based on the estimated mean and SD of the 2011–2019 within‐season resight data.

### Model fitting

Posterior samples were drawn using Markov Chain Monte Carlo (MCMC) simulations in JAGS v. 4.3.0 (Plummer, 2003), called using the “rjags” package in program R v. 4.0 (Plummer, 2016; R Core Team, 2020). Models were implemented with an adaptive phase of 1000 iterations, and inferences were drawn from another 35,000 samples from each of three MCMC chains. We incorporated vague priors on all parameters with the exception of $p^{\left(r\right)}$ at the low‐elevation plot at the trailing edge (described above) and the density‐dependence parameter $\beta_{2}^{\left(\gamma \right)}$ on per‐capita recruitment. We constrained the prior for this parameter to only consider negative density dependence, as breeding‐season Allee effects do not seem to be important in this system (Merker & Chandler, 2021). Regression parameters for per‐capita recruitment, apparent survival, capture probability, and detection probability were modeled as arising from Gaussian distributions with mean 0. Parameters for aging females into specific age categories on capture (k) and the age ratio between SY and ASY birds (τ) were modeled as arising from uniform distributions, while the bounding parameter on gamma (α) was modeled as arising from an exponential distribution. See Lewis et al. (2022) for more details. Model convergence was assessed by visually inspecting the MCMC chains. All point estimates reported in the “*Results*” section are presented as median estimates and 95% credible intervals (CrI).

### Population viability and forecasting

We used statistical forecasting to assess population viability through 2040 (Clark et al., 2001; Desforges et al., 2018; Hooker et al., 2020; Howell et al., 2020). Statistical forecasting accounts for process variance (i.e., demographic stochasticity and environmental change) as well as parameter uncertainty (Zylstra & Zipkin, 2021). We forecasted dynamics at each study plot using the temporal‐trend and climate‐trend models. For climate models, we simulated future climate variables in each year of the forecasts based on the 2002–2019 trend in the climate variable at each range position. Specifically, we used the estimated mean $\left(\mu^{\left(w_{\text{trend}}\right)}\right)$ and SE $\left(\sigma^{\left(w_{\text{trend}}\right)}\right)$ of the time‐trend effect from the linear model assessing long‐term changes in climate at each range position (see *Climate data*). We assumed that future trends in climate variables would be similar across study plots at each range position. The climate in future years was simulated by: $w_{t+1}∼\text{Norm}\left(w_{t}+\mu^{\left(w_{\text{trend}}\right)},\sigma^{\left(w_{\text{trend}}\right)}\right)$.

Forecast distributions were computed using the MCMC samples. For each posterior sample, we used the time‐or‐climate‐trend in γ and $\phi_{j}$ to forecast individual recruitment and survival for each year between the last year of data at the plot and 2040. We initialized forecasts based on the age $(a_{i,\text{lastyear}}$) and alive/dead state ($z_{i,\text{lasteyear}}$) of each individual 1:M in the last year of data at the plot. We augmented this dataset with an additional 2400 possible individuals ($M_{\text{future}}$) which could enter the local population during forecasting. Entry and exit of individuals from the local population were simulated for each year of the forecasts similar to above, except that the probability of entering the local population in a future year was modeled as:

(6) $$\mathrm{Pr}\left(b_{i}=t_{\text{future}}\right)=\frac{E\left(n_{1,t_{\text{future}}}\right)}{M_{\text{future}}}.$$

We assessed local extinction risk at each study plot as the percentage of forecasts which had reached 0 density in 2040.

## RESULTS

### Trailing edge

**FIGURE 5:**
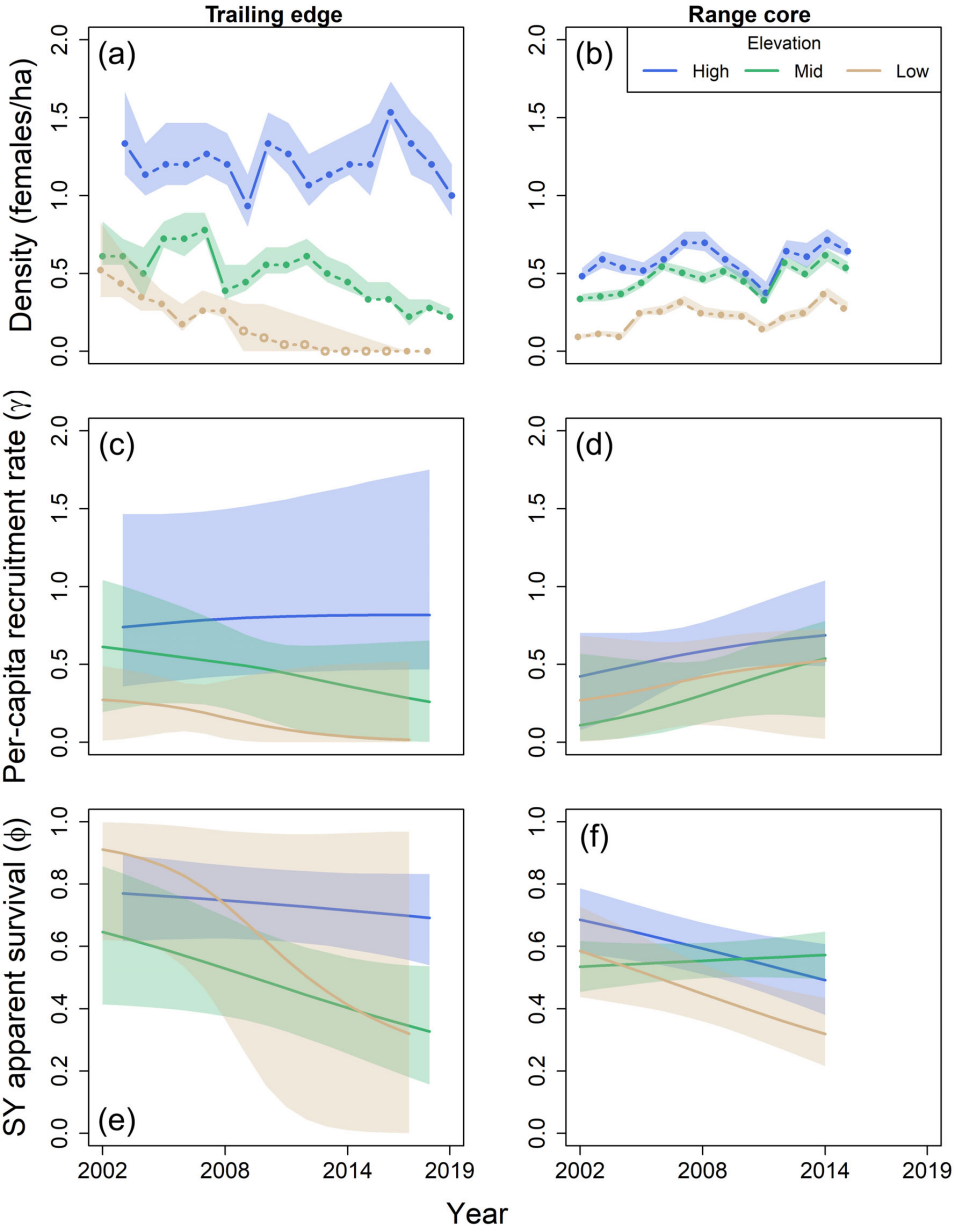
Estimates of annual density of female black‐throated blue warblers (*Setophaga caerulescens*) (a, b) and predicted trends in per‐capita recruitment (c, d) and second‐year (SY) apparent survival (e, f) from the Bayesian hierarchical analysis, 2002–2019. After‐second‐year apparent survival showed a similar temporal trend, but the intercept was lower for all study plots. Model estimates are shown for the trailing edge (North Carolina, a, c, e) and range core (New Hampshire, b, d, f) of the breeding range. For the low‐elevation plot at the trailing edge, filled circles represent years when the plot was surveyed. Mean values and 95% credible intervals (shaded) are shown.

Local population density was positively associated with elevation at the trailing edge (Figure 5a). Density fluctuated annually but did not show any long‐term trend at the high‐elevation plot at the trailing edge; however, local population declines occurred at both the low‐ and mid‐elevation plots (Figure 5a). Local population density at the low‐elevation plot declined steadily from 12 females in 2002 (95% CrI, 8–19) to six females in 2008 (CrI 5–8). The monitoring effort ceased at the low‐elevation plot after 2008 due to low female density and associated difficulty in studying demography (Stodola et al., 2013), but follow‐up surveys in 2017 and 2018 detected no female black‐throated blue warblers. In 2018, we detected one male black‐throated blue warbler at the low‐elevation plot. This bird appeared to be an SY based on a prominent brown tinge on the flight feathers, and thus was likely to have been recruited that year. The bird was observed traveling around a broad area broadcast singing, and no evidence of a paired female was detected. The local population at the mid‐elevation plot was stable until about 2012, after which it declined from 11 females in 2012 (CrI 10–13) to four females in 2019 (CrI 4–5).

**FIGURE 6:**
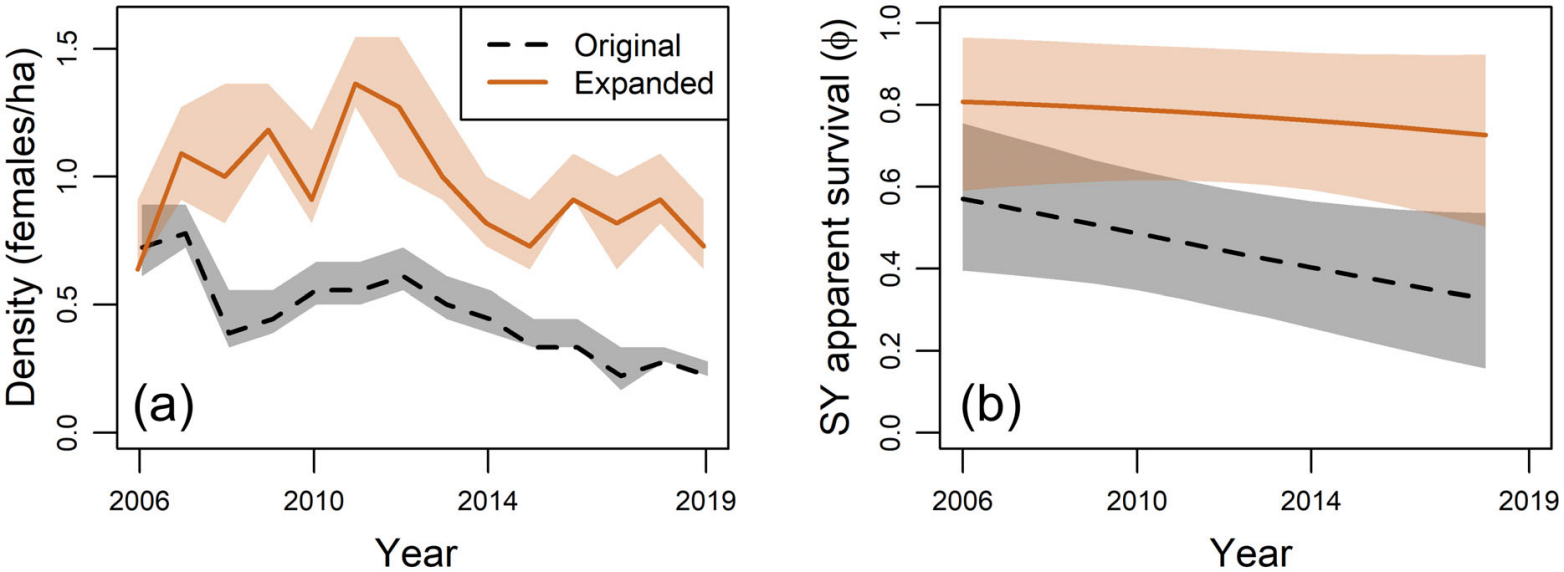
Estimates from the Bayesian hierarchical analysis of annual density (a) and predicted trends in second‐year (SY) apparent survival (b) of female black‐throated blue warblers (*Setophaga caerulescens*) breeding on the original (dashed, black) and expanded (solid, orange) sections of the mid‐elevation plot at the trailing edge in North Carolina. Sections of the plot differed in habitat quality, with the expanded section representing high‐quality habitat and the original section representing low‐quality habitat (see *Study sites* for more details). After‐second‐year apparent survival showed a similar temporal trend, but the intercept was lower for both sections of the plot. Mean values and 95% credible intervals (shaded) are shown.

Temporal trends in demographic rates differed among study plots at the trailing edge (Lewis et al., 2022). Although both per‐capita recruitment rate and apparent survival declined during the period of population decline at the low‐elevation plot (Figure 5c,e), uncertainty in estimates was high (recruitment: −1.2, CrI −6.62 to 3.09; survival: −1.15, CrI −3.28 to 0.48). The exact demographic drivers of extirpation at the low‐elevation plot are therefore unclear. This high uncertainty at the low‐elevation plot was, in part, driven by the low sample size, as the plot was only sampled intensively for 7 years. Furthermore, only a few individuals were estimated to be present in the local population in the later years, such that the loss or gain of even one individual had a substantial effect on per‐capita demographic rates. Local population decline at the mid‐elevation plot was largely driven by declining apparent survival (Figure 5e), although CrIs overlapped zero (−0.34, CrI −0.75 to 0.02). During the period of local population decline (2012–2019), 1–3 SY females were recruited to the plot each year, but few returned to breed in subsequent years. In contrast with this temporal trend on the original section of the mid‐elevation plot, breeding density and apparent survival only showed minor declines in the expanded section (Figure 6).

**FIGURE 7:**
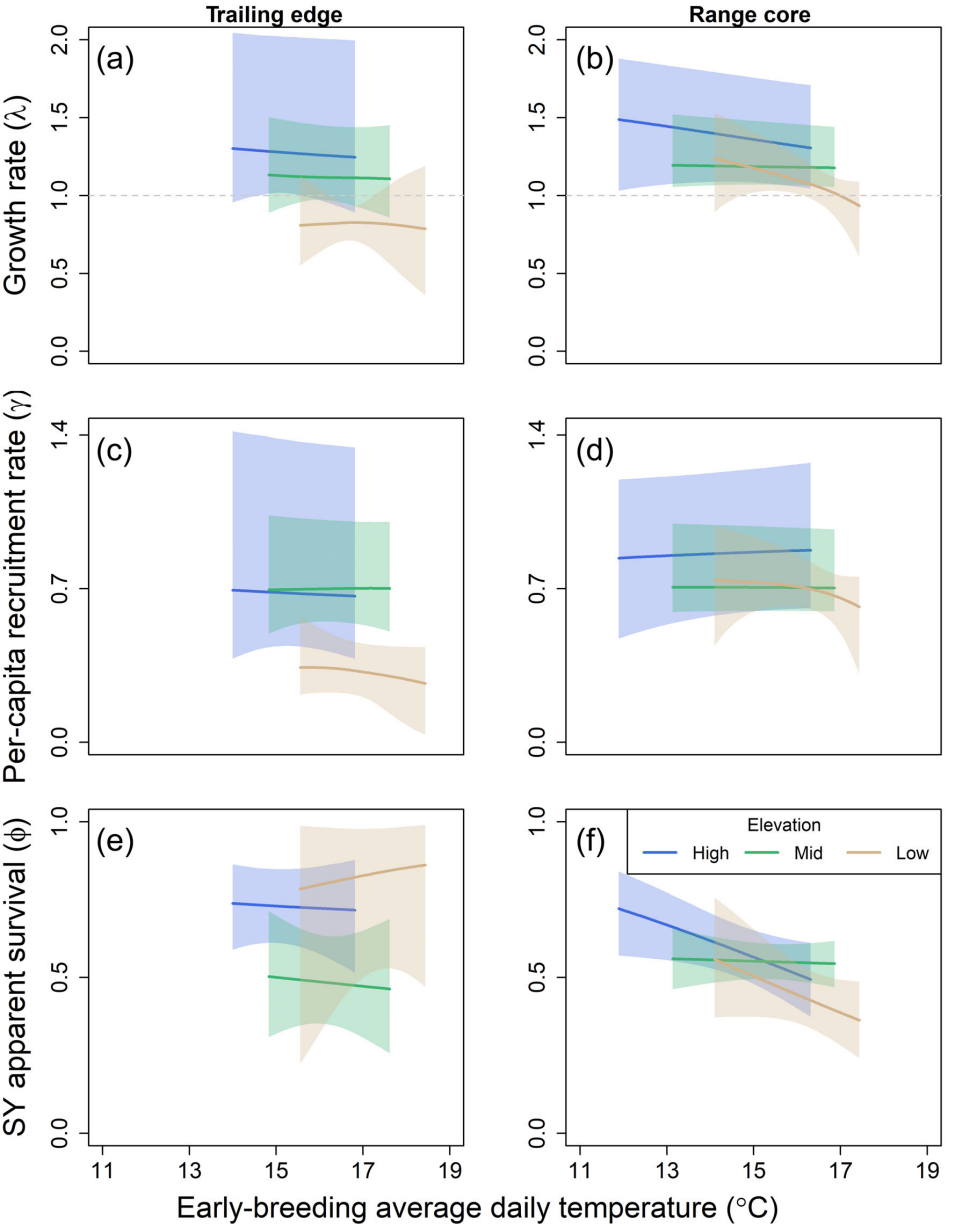
Predicted effect of early‐breeding temperature (average lay date to average fledge date of first broods) on the finite population growth rate (a, b), per‐capita recruitment (c, d), and second‐year (SY) apparent survival (e, f) of female black‐throated blue warblers (*Setophaga caerulescens*) at the trailing edge (North Carolina, a, c, e) and core (New Hampshire, b, d, f) of the breeding range. Figures show the population growth or demographic rate from year *t* to *t* + 1 in relation to early‐breeding temperature in *t*. Effects of early‐breeding temperature on per‐capita recruitment were predicted at a density of 0.3 females/ha. After‐second‐year apparent survival showed a similar trend, but the intercept was lower for all study plots. Mean values and 95% credible intervals (shaded) are shown. See *Methods* for more details.

Model uncertainty was high when estimating climate effects on per‐capita recruitment and apparent survival at the trailing edge. Uncertainty in parameter estimates was especially high at the low‐elevation plot with no strong effect of either early‐breeding temperature or annual precipitation (Figure 7a,c,e). We found no effect of early‐breeding temperature over the entire study period at the mid‐elevation plot at the trailing edge (Figure 7a,c,e); however, population declines were not observed in this plot until 2012. We re‐ran the hierarchical model at the mid‐elevation plot, specifying a separate trend effect of early‐breeding temperature on demographic rates for the periods before and after 2012. Early‐breeding temperature negatively affected apparent survival during the period of local population decline (2012–2019), although CrIs overlapped zero (−0.56, CrI −0.94 to 0.12). We used a linear model to determine whether temperatures increased more during the period of population decline than during the period of stability. The model was fitted using an interaction between year (trend effect) and period (before and after 2012) to explain variation in early‐breeding temperature. The warming trend in this plot was not significantly different before and after 2012 (interaction term 95% CI: −0.16 to 0.52), indicating that the differential response to early‐breeding temperature after 2012 was not caused by differences in warming trends. We did not observe a strong effect of annual precipitation on demographic rates at the mid‐elevation plot, nor did we observe a strong effect of either climate variable at the high‐elevation plot (Figure 7a,c,e).

### Range core

Local population density was positively associated with elevation at the range core, although female density at the range core was approximately half the observed density at the high‐elevation study plot at the trailing edge (Figure 5b). Female density remained relatively stable over time (no long‐term trend) at the high‐elevation plot at the range core, but increased over time at the low‐ and mid‐elevation plots (Figure 5b). Between 2002 and 2015, the local population size increased from 9 (CrI 8–11) to 27 (CrI 26–31) females at the low‐elevation plot and 42 (CrI 40–46) to 67 (CrI 62–72) females at the mid‐elevation plot. Despite the stable or increasing density, apparent survival declined over time at both the low (−0.37, CrI −0.67 to −0.10) and high (−0.27, CrI −0.50 to −0.05) elevation plots (Figure 5f). The declining apparent survival at the high‐elevation plot was, at least partially, offset by a trend for increasing per‐capita recruitment, although CrIs overlapped zero (Figure 5d; 0.61, CrI −0.77 to 2.16).

**FIGURE 8:**
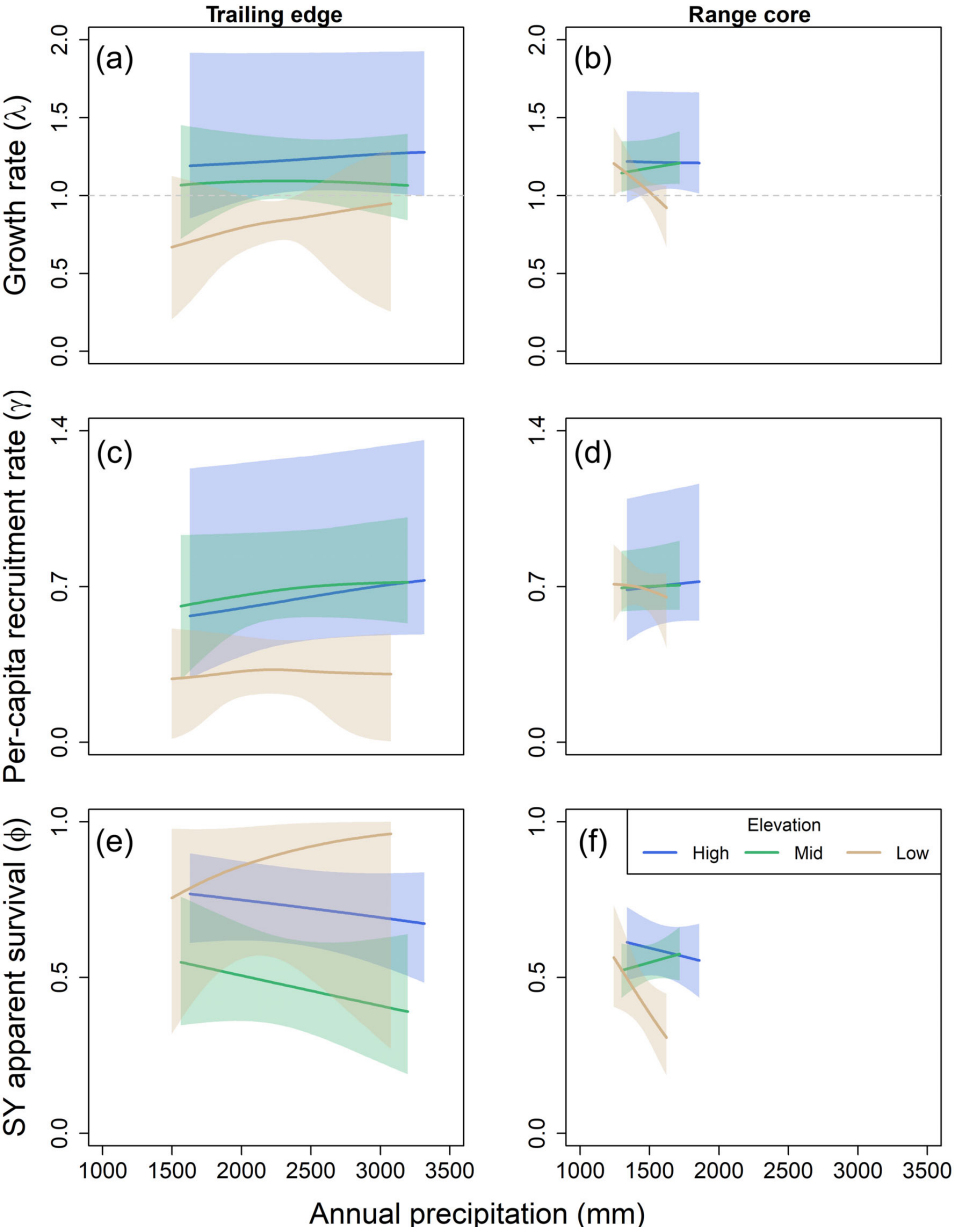
Predicted effect of annual precipitation on the finite population growth rate (a, b), per‐capita recruitment (c, d), and second‐year (SY) apparent survival (e, f) of female black‐throated blue warblers (*Setophaga caerulescens*) at the trailing edge (North Carolina, a, c, e) and core (New Hampshire, b, d, f) of the breeding range. Figures show the population growth or demographic rate from year *t* to *t* + 1 in relation to annual precipitation in *t*. Effects of annual precipitation on per‐capita recruitment were predicted at a density of 0.3 females/ha. After‐second‐year apparent survival showed a similar trend, but the intercept was lower for all study plots. Mean values and 95% credible intervals (shaded) are shown. See *Methods* for more details.

The strongest climate effects on demographic rates at the range core occurred at the low‐elevation plot, where local population growth was negatively associated with early‐breeding temperature (Lewis et al., 2022). This relationship was driven by a negative effect of early‐breeding temperature on apparent survival, although CrIs overlapped zero (Figure 7b,e; 0.25, CrI −0.63 to 0.07). Early‐breeding temperature also was negatively related to apparent survival at the high‐elevation plot (Figure 7e; apparent survival −0.26, CrI −0.51 to −0.02); however, this pattern was mostly driven by a few years with high early‐breeding temperatures and low apparent survival. Apparent survival and local population growth rate were negatively correlated with annual precipitation in the previous year at the low‐elevation plot at the range core (Figure 8b,f; −0.38, Cr −0.65 to −0.01).

### Statistical forecasting

**FIGURE 9:**
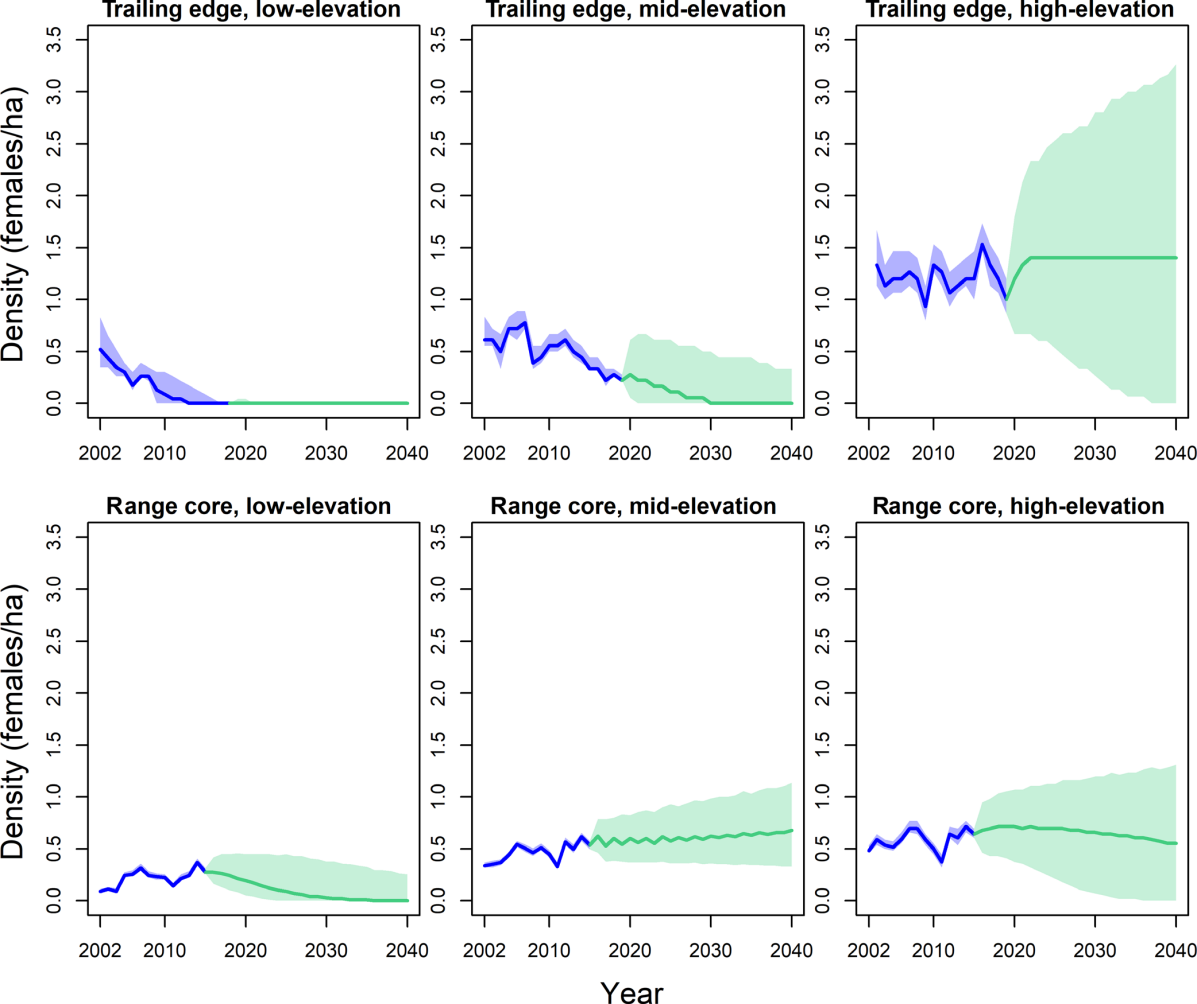
Forecasts of female black‐throated blue warbler (*Setophaga caerulescens*) breeding density through 2040 at the trailing edge (North Carolina, top row) and core (New Hampshire, bottom row) of the breeding range under current temporal trends in population dynamics. Estimates over the course of the study period (dark blue) and forecasts (green) are shown. Mean (solid line) and 95% credible intervals (shaded) are shown. See *Population viability and forecasting* for more details.

Statistical forecasting with the temporal model projected that density would remain relatively stable through 2040 at four of the six study plots (Figure 9). Specifically, density was projected to remain relatively stable at the mid‐elevation plot at the range core and at both high‐elevation plots, while density at the low‐elevation plot at the trailing edge was projected to remain at 0. In contrast, local extinction risk was projected to be 87% and 68% in 2040 at the mid‐elevation plot at the trailing edge and the low‐elevation plot at the range core, respectively. The local extinction risk at the low‐elevation plot at the range core was driven by the declining apparent survival.

**FIGURE 10:**
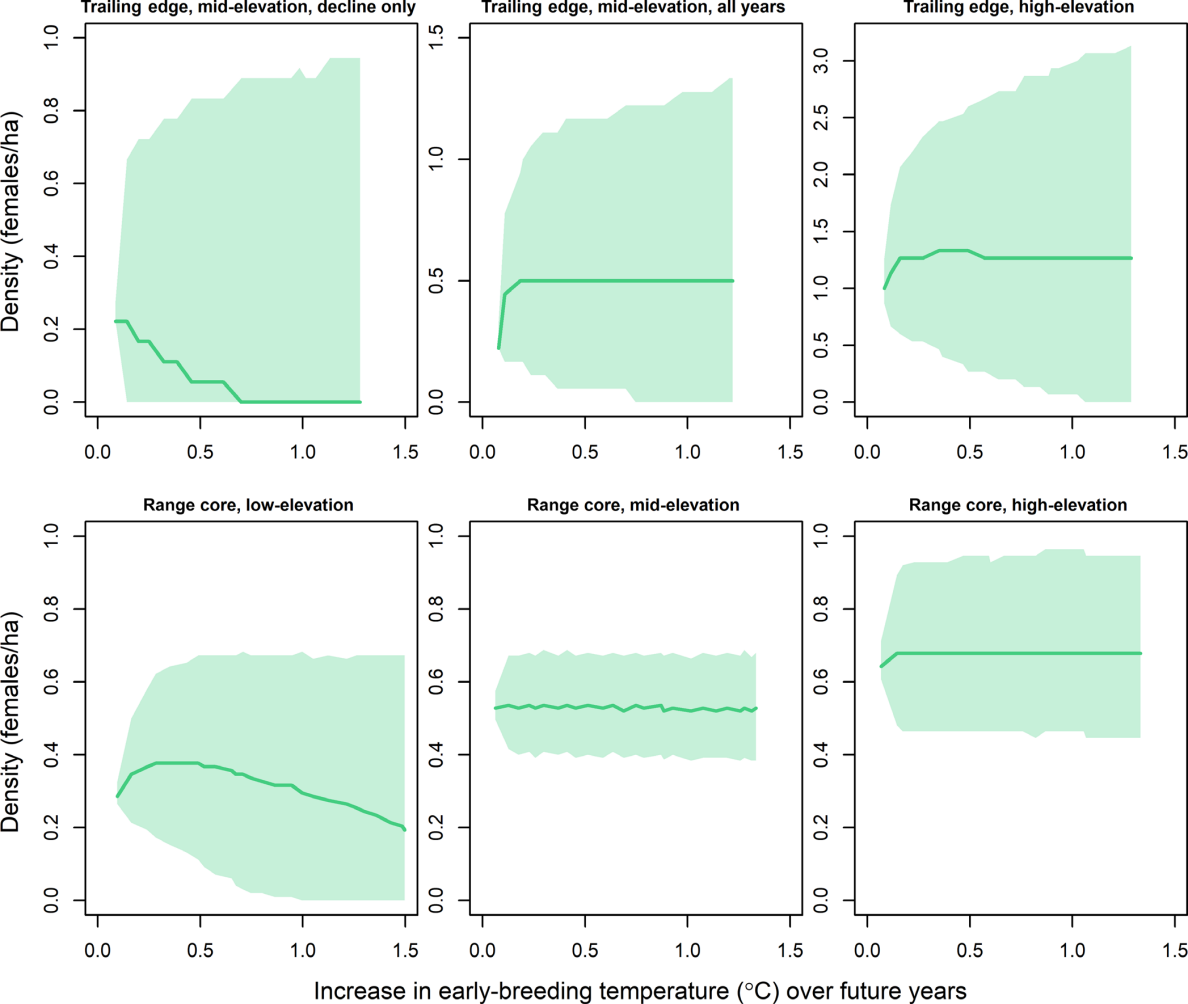
Forecasts of female black‐throated blue warbler (*Setophaga caerulescens*) breeding density in relation to projected changes in average daily early‐breeding temperature from the last year of the study at each plot through 2040. Forecasts are shown for the trailing edge (North Carolina, top row) and core (New Hampshire, bottom row) of the breeding range under current temperature trends in population dynamics. Mean (solid line) and 95% credible intervals (shaded) are shown. Future increases in early‐breeding temperatures were simulated at each study plot based on the 2002–2019 trend in temperature at each range position. Forecasts are not shown for the low‐elevation plot at the trailing edge because the density in this plot had already fallen to 0 by the end of the study. Forecasts for the mid‐elevation plot at the trailing edge were performed under dynamics estimated over the entire study period (2002–2019, top middle) and under dynamics estimated only during the period of local population decline (2012–2019, top left). See *Population viability and forecasting* for more details.

**FIGURE 11:**
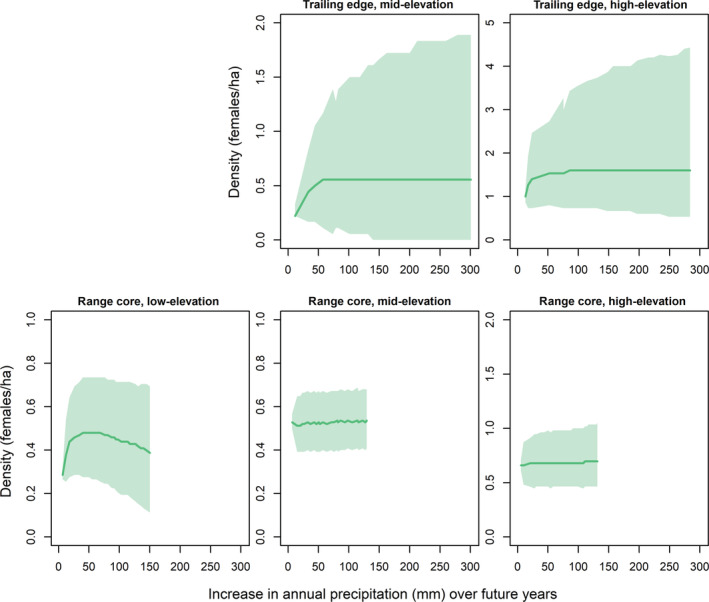
Forecasts of female black‐throated blue warbler (*Setophaga caerulescens*) breeding density in relation to projected changes in annual precipitation from the last year of the study at each plot through 2040. Forecasts are shown for the trailing edge (North Carolina, top row) and core (New Hampshire, bottom row) of the breeding range under current precipitation trends in population dynamics. Mean (solid line) and 95% credible intervals (shaded) are shown. Future increases in annual precipitation were simulated at each study plot based on the 2002–2019 trend in precipitation at each range position. Forecasts are not shown for the low‐elevation plot at the trailing edge because density in this plot had already fallen to 0 by the end of the study. See *Population viability and forecasting* for more details.

The higher degree of uncertainty in the climate models compared to the temporal models generally led to high uncertainty when forecasting population dynamics based on projected future climate (Figures 9, 10, 11). Early‐breeding temperatures were projected to increase by 1–1.5°C by 2040 at the study plots (Figure 10). Forecasting over projected temperature increases resulted in generally similar patterns compared with forecasting over future years, with warming temperatures projected to lead to population declines at the low‐elevation plot at the range core but having little effect at the high‐elevation plots or the mid‐elevation plot at the range core (Figure 10). Warming temperatures were projected to have little effect on local population density at the mid‐elevation plot at the trailing edge when forecasting with dynamics estimated over the entire survey period (Figure 10, top middle). Forecasting with dynamics estimated only during the period of local population decline in this plot, however, led to local population declines with warming temperatures (Figure 10, top left). The mid‐elevation plot at the trailing edge, under dynamics during local population decline, and the low‐elevation plot at the range core were projected to have local extinction risks of 74% and 12%, respectively, in response to warming temperatures by 2040. In contrast, projected increases in annual precipitation through 2040 were forecast to have only minor effects on population density at most study plots (Figure 11).

## DISCUSSION

Our results suggest that climate change is affecting local black‐throated blue warbler populations at both the low‐latitude and low‐elevation edges of the breeding range. Long‐term demographic trends supported both climate‐change hypotheses: breeding density and demographic rates were correlated with the climate gradient at both range positions, and the strongest negative effects occurred at the lowest, warmest breeding areas, especially at the trailing edge. The local population at the lowest, warmest elevation plot in North Carolina became extirpated during the course of the study, and the local population at the mid‐elevation plot was projected to be at risk of extirpation by 2040. While breeding density was stable or increasing at the New Hampshire study plots over the course of the study, the local population at the low‐elevation plot there was projected to decline over the next decade. The projection for local population declines in New Hampshire to occur first at the warmest breeding habitat is consistent with our climate‐change hypotheses and suggests that even populations in the geographic core of the range may be affected by climate change (Boakes et al., 2018; Channell & Lomollno, 2000).

Model estimates for climate effects exhibited a greater degree of uncertainty than for the temporal patterns of local population declines, so our inferences about climate effects on demographic rates and population dynamics must be taken with a degree of caution. Climate change affects populations over long time periods, so the high uncertainty when estimating climate effects may be, at least partially, because the study duration at some plots was not long enough to adequately detect climate effects on demography. The low‐elevation plot at the trailing edge was only intensively sampled for 7 years, which likely contributed to the lack of precision in parameter estimation at this plot. Similarly, local population declines and negative effects of temperature on demographic rates were not observed at the mid‐elevation plot at the trailing edge until the last 8 years of the study. Despite the uncertainty in parameter estimation, the effects of early‐breeding temperature were generally consistent with our climate‐change hypotheses: we found a general trend for temperature to be negatively associated with demographic rates and population growth at most study plots experiencing observed or projected local population declines. Furthermore, warming temperatures through 2040 were projected to lead to local population declines at these study plots. The exception was the low‐elevation plot at the trailing edge, where the low local population size and few years of data resulted in imprecise demographic estimates. We are therefore unable to draw strong inferences about the effects of climate on local population declines at this study plot. Despite the equivocal results at the low‐elevation plot at the trailing edge, the general trend for local population declines to be most pronounced at low elevations, especially at the trailing edge, and the negative effect of temperature at other study plots suggests that local population declines were associated with climate change.

Our study was not designed to assess the mechanisms through which climate change may be affecting populations. Climate change may have had direct effects on black‐throated blue warblers, such as physiological stress causing egg or nestling mortality (Cunningham et al., 2013; Grant, 1982), but the main effects are likely to be indirect through biotic interactions (Cahill et al., 2013; Thomas, 2010). For example, climate change may be indirectly affecting populations of black‐throated blue warblers by affecting changes in the phenology, activity patterns, or distribution of prey species, nest predators, or competitors (Cox et al., 2013; Cunningham et al., 2013; Myers et al., 2009; Stenseth et al., 2015; van de Pol et al., 2010; Visser et al., 2006). Parameter estimates were less precise when modeling climate effects than when modeling temporal effects, supporting the hypothesis that climate effects on black‐throated blue warbler populations are largely indirect. The process of local population decline in black‐throated blue warblers is likely to be complex, but our results suggest that climate change is likely to be a major cause of declines at the low‐latitude and low‐elevation range edges in this species.

Our results are consistent with the rapidly growing body of literature documenting trailing edge range contractions, likely as a result of climate change (Coristine & Kerr, 2015; Moritz et al., 2008; Rushing et al., 2020; Taheri et al., 2020; Wilson et al., 2005). Local population declines in North Carolina were first observed at the lowest, warmest plot and were not observed at higher, cooler elevations until later in the study period. This pattern is expected if the factors setting warm‐edge range limits have gradually shifted further upslope in response to climate change. Lower‐elevation local population declines in North Carolina support the hypothesis that trailing edge populations breeding at the lowest, warmest elevations are near the ecological tolerance of the species (Brown, 1984; Holt et al., 2005). Furthermore, the general trend of temperature negatively affecting demographic rates at the low‐latitude and lower‐elevation study plots suggests that the factors setting the low‐elevation range boundary for this species are directly or indirectly affected by climate. Local populations of black‐throated blue warblers near the warm‐edge range boundary at low latitudes and low elevations are therefore likely to be particularly vulnerable to climate change, pushing them past their ecological tolerance (Deutsch et al., 2008), and potentially leading to local population declines, extirpation, and range contractions.

Local population declines at the trailing edge may be affected by numerous processes, but our results suggest that climate change is likely to be one of the most influential. For example, the southern Appalachians have also experienced the extensive loss of a major canopy tree species, eastern hemlock (*Tsuga canadensis*) due to the introduced hemlock woolly adelgid (*Adelges tsugae*) (Webster et al., 2012). A previous analysis of the 2002–2008 trailing edge, black‐throated blue warbler data concluded that population declines at the low‐elevation plot were attributable to hemlock loss, because black‐throated blue warblers used hemlock as a secondary nesting substrate at the low‐elevation plot but not at the higher elevations (Stodola et al., 2013). While hemlock loss likely contributed to declines at the low‐elevation plot, recent black‐throated blue warbler local population declines at the mid‐elevation plot in North Carolina suggest that hemlock loss is unlikely to be the sole driver of declines at the trailing edge. While hemlock was almost completely extirpated from the area by 2011 (Webster et al., 2012), the low‐elevation plot still contains enough cover of the preferred nesting substrate, rhododendron, to support several black‐throated blue warbler breeding pairs (W. Lewis, personal observation). Moreover, the mid‐elevation plot historically had a low density of hemlock, and thus was less affected by its loss (Appendix S1: Figure S1). Local population declines at the mid‐elevation plot are well explained by climate‐change hypotheses but not by the hemlock loss hypothesis. We do not have long‐term vegetation data from the low‐elevation plot at the trailing edge to assess how vegetation has changed in this plot in relation to hemlock loss, but decreasing hemlock canopy cover has led to increased rhododendron cover in the surrounding area at the trailing edge (Ford et al., 2012, Appendix S1: Figure S1). Understory deciduous leaf cover is a good predictor of black‐throated blue warbler habitat quality (Holmes et al., 1996; Kaiser et al., 2015; Rodenhouse et al., 2003; Steele, 1993); however, deciduous sapling density did not change over the study period at the higher‐elevation study plots at the trailing edge despite widespread hemlock loss at the high‐elevation plot (Appendix S1: Figure S1). Furthermore, populations of many bird species are declining at the trailing edge of their range in the southern Appalachians (Sauer et al., 2017), although many of these species do not utilize hemlock. Hemlock loss at the low‐elevation study plot at the trailing edge may have exacerbated local population declines, but climate change appears most likely to be the primary driver of black‐throated blue warbler population declines over the broader study area in North Carolina.

Although the pattern of local population declines was generally consistent with our climate‐change hypotheses, the demographic drivers of declines varied between study plots. Extirpation at the low‐elevation plot in North Carolina was associated with declining recruitment and apparent survival, but the model could not differentiate between the two because of high uncertainty. The age ratio became very heavily skewed toward older birds during the period of decline in this plot, suggesting that the declining recruitment of young birds may be the primary driver of local population declines (Stodola et al., 2013). In contrast with the low‐elevation plot at the trailing edge, observed and predicted declines for the mid‐elevation plot at in North Carolina and the low‐elevation plot in New Hampshire were driven by declining adult apparent survival. Previous studies have found that changes in both recruitment (Neate‐Clegg et al., 2021; Waite & Strickland, 2006) and apparent survival (Srinivasan & Wilcove, 2021) can be the primary drivers underlying avian trailing edge population declines. The heterogeneous demographic drivers of local population declines observed between study plots in our study, and across previous studies, suggests that response to climate change is likely to be complex and affected by factors such as species traits, the severity of climate change experienced by the local population, and proximity to range edges (Srinivasan & Wilcove, 2021; Tingley et al., 2012).

True survival is frequently inseparable from permanent emigration in ecological studies; however, we hypothesize that population declines in our study system were driven primarily by increasing permanent emigration at many, but not all, study plots. Apparent survival declined precipitously in the original, largely poor‐quality section of the trailing edge, mid‐elevation plot despite remaining relatively stable in the expanded, higher quality section. True annual survival would be unlikely to vary drastically between females breeding in adjacent sections of a study plot. Thus, population declines in the original section likely arose from increasing rates of permanent emigration. Emigration rates of black‐throated blue warblers are negatively correlated with habitat quality (Cline et al., 2013; Holmes et al., 1996), explaining why permanent emigration would be increasing in the poor‐quality original section of the plot but not in the higher quality expanded section. Several SY females were recruited to the poor‐quality original section of the plot annually, an expected pattern if younger, competitively‐subordinate birds are competitively‐excluded from higher quality habitats (Rodenhouse et al., 1997, 2003). A similar mechanism may have occurred at the low‐elevation plot in New Hampshire. Habitat quality is positively correlated with elevation at the range core (Cline et al., 2013), and adults engaging in breeding dispersal generally shift to higher quality territories (Holmes et al., 1996; Rodenhouse et al., 2003). Although we do not know the exact mechanisms, increasing rates of permanent emigration may have arisen if warming temperatures led to declining local breeding conditions (Cline et al., 2013; Doligez et al., 2002; Greenwood & Harvey, 1982). Feather stable isotopes of black‐throated blue warblers suggest that adults in the southern Appalachians largely exhibit high fidelity to the same elevation range in which they bred in the previous year (Graves et al., 2002); however, this finding does not contradict our emigration hypothesis. High adult site fidelity is likely to be the norm in this system, especially in high‐quality habitats and following years of good breeding conditions, with adult emigration being less common and a conditional response to poor breeding conditions. Furthermore, birds may only disperse short distances when emigrating and thus stay within the same general elevational range as in previous years. We therefore suggest that adult emigration from poor‐quality habitats may be a method by which birds can behaviorally respond to adverse breeding conditions at the local scale.

Our results suggest that black‐throated blue warbler demography is generally more sensitive to changes in temperature than to changes in precipitation. Variation in annual precipitation had little effect on warbler demographic rates and population growth except at the low‐elevation plot at the range core, where apparent survival declined in wetter years. Heavy rainfall events at the range core have previously been found to increase rates of nest failure and nestling mortality (Rodenhouse & Holmes, 1992), which may lead to adult emigration following reproductive failure (Cline et al., 2013; Greenwood & Harvey, 1982). The reason why this effect would only be observed at the low‐elevation plot is not clear. Regardless of the mechanisms driving this pattern at the low‐elevation plot at the range core, local population declines in black‐throated blue warblers appear driven more by changes in temperature than by changes in precipitation. We may not have found a strong effect of precipitation because we did not model the actual precipitation variables or time periods during which precipitation affects demography (Tamburini et al., 2013; van de Pol & Cockburn, 2011). For example, productivity may be more affected by variation in annual precipitation and extreme precipitation events rather than by total annual precipitation (Martin et al., 2017; Sillett et al., 2004), or variation in precipitation may have a time‐lagged effect on populations (Pearce‐Higgins et al., 2015). A more detailed study is needed to determine the effects of changing precipitation on black‐throated blue warbler population dynamics.

The observed demographic trends in black‐throated blue warblers generally conformed to our climate‐change hypotheses, although our inferences could be strengthened through the replication of study plots across latitudes and elevations. The cost and effort associated with long‐term demographic monitoring, however, are generally prohibitive for broad‐scale demographic monitoring. Future work should attempt to use new methods allowing for the integration of intensive demographic data with other sources of information that are less informative about demographic processes but easier to collect (Chandler et al., 2018). Despite the small number of study plots, several lines of evidence suggest that results from our plots are representative of dynamics across the broader study areas. First, recruitment at the range core is positively correlated with productivity in the previous year (Holmes et al., 1992; Sillett et al., 2000; Sillett & Holmes, 2005). Very few birds are recruited at the study plot at which they were fledged (Nagy & Holmes, 2005), so this positive relationship suggests, at least at the range core, that demographics at the study plots are reflective of the broader area. Second, the observed pattern of local population declines at the trailing edge, but not at the range core, is consistent with regional trends from the Breeding Bird Survey (Sauer et al., 2017). Breeding Bird Survey data indicate that regional populations in New Hampshire are relatively stable (Sauer et al., 2017), perhaps suggesting that adults at the range core may be emigrating upslope from lower elevations in response to warmer temperatures (Van Tatenhove et al., 2019). Regional population declines in the southern Appalachians (Sauer et al., 2017), however, suggest that changes in adult emigration, and perhaps recruitment, observed at our study plots may represent broader‐scale regional population declines at the trailing edge. Point count data collected across the trailing edge study area also suggest that the center of distribution of black‐throated blue warblers has been steadily moving upslope, with local extinction rates being positively associated with temperature (Merker, 2017). Furthermore, populations of many bird species are declining and abandoning low‐elevation breeding habitats at the trailing edge of their range in the southern Appalachians (Merker, 2017; Sauer et al., 2017). For at least one of these trailing edge species, the Canada warbler (*Cardellina canadensis*), apparent survival is also lowest at the lower, warmer elevations in the southern Appalachians (Chandler et al., 2018). This could indicate that many trailing edge species may respond to warming local temperatures through emigration, and that the demographic results from black‐throated blue warblers may be used in mechanistic models to predict the effects of climate change for a range of species in the southern Appalachians.

## CONCLUSIONS

We documented local population declines and range contractions of black‐throated blue warblers at the low‐latitude and low‐elevation edges of the breeding range. Our results suggest that local population declines were frequently associated with increasing average temperatures during the early‐breeding season. The demographic drivers of decline differed between study plots, but emigration away from poor‐quality habitats may be a common behavioral response to warming temperatures. Understanding where adults settle after emigrating away from the study plots is a priority for future research, but tracking the dispersal movements of small birds across broad areas of contiguous forest is difficult. One avenue may be to monitor the number of unmarked adult birds immigrating to high‐elevation breeding habitats, as this should increase if birds from low‐elevation habitats are emigrating to higher quality habitats at high elevations (Holmes et al., 1996). Upslope emigration of adults may be unlikely given the high site fidelity observed in Graves et al. (2002), although it is possible that rates of adult upslope emigration may have increased over the last few decades if warming temperatures at low elevations have pushed past the ecological tolerance of this species. Additionally, our results suggest that early‐breeding temperatures were associated with local population declines in black‐throated blue warblers, but more study is needed to determine the proximate mechanisms, such as changes in vegetation, competitors, or food resources, through which warming temperatures may be causing changes in demographic rates and population dynamics.

Our results suggest that climate change on the breeding grounds is a dominant driver of trailing edge declines, but population dynamics of migratory species are governed by threats encountered during the entire annual cycle (Runge & Marra, 2005; Sillett et al., 2000). Black‐throated blue warblers from the trailing edge and the range core overwinter in broadly different areas of the Caribbean (Rubenstein et al., 2002), so habitat loss or other threats encountered during the nonbreeding season may have contributed to breeding‐ground declines. While our study was not designed to assess how threats encountered throughout the annual cycle interact to drive population dynamics, several lines of evidence suggest that conditions on the breeding grounds have been a major driver of the observed local population declines. First, we observed a general trend of early‐breeding temperatures on the breeding grounds to be negatively associated with demographic rates and projected future population trends. Furthermore, populations of many species of birds are declining at the trailing edge of their breeding ranges in the southern Appalachians (Sauer et al., 2017), despite wintering in broadly different areas of the Neotropics. Taken together, our results strongly support the hypothesis that climate change on the breeding grounds contributes to local population declines in black‐throated blue warblers, but further research is needed to determine how climate change on the breeding grounds interacts with threats encountered during other seasons to shape population dynamics throughout the annual cycle (e.g., Hallworth et al., 2021; Rushing et al., 2017; Sillett et al., 2000). Understanding the drivers of range contractions of migratory birds in montane regions is critically important. Ongoing climate change may shift the low‐elevation range limit further upslope; however, populations in many areas are already concentrated at the highest elevations, with little potential for further elevational expansion.

## CONFLICT OF INTEREST

The authors declare no conflict of interest.

## Supporting information


Appendix S1

## Data Availability

Data, JAGS model, R code, and tables of parameter and realized estimates for Bayesian hierarchical models estimating population dynamics and demographic rates of black‐throated blue warblers (*Setophaga caerulescens*) (Lewis et al., 2022) are available on Zenodo at https://doi.org/10.5281/zenodo.7087841.
