# Supplementary material for: Climate‐mediated population dynamics of a migratory songbird differ between the trailing edge and range core
Source: Ecol Monogr. 2023 Jan 4;93(1):e1559. doi: 10.1002/ecm.1559 (PMC10078169; doi:10.1002/ecm.1559)
Supplement: Supplementary file 1 — Appendix S1 [file ECM-93-0-s001.pdf]

Supporting Information. Lewis. W. B., R. J. Cooper, R. B. Chandler, R. W. Chitwood, M. H. Cline, M. T. Hallworth, J. L. Hatt, J. Hepinstall-Cymerman, S. A. Kaiser, N. L. Rodenhouse, T. S. Sillett, K. W. Stodola, M. S. Webster, and R. T. Holmes. Climate-mediated population dynamics of a migratory songbird differ between the trailing edge and range core. Ecological Monographs.

Appendix S1: Supporting tables and figures

Table S1. USDA Forest Service climate stations to assess the effects of temperature and precipitation on population dynamics of black-throated blue warblers (*Setophaga caerulescens*) breeding at the trailing edge of the range at the Coweeta Hydrologic Laboratory in North Carolina (Trailing) and core of the range at the Hubbard Brook Experimental Forest in New Hampshire (Core).

Range Position	Climate Variable	Station ID	Elevation	Latitude	Longitude
Trailing	Temp	CS17	884	35.0454	-83.4374
Trailing	Temp	CS28	1189	35.0466	-83.4650
Trailing	Temp	CS77	1389	35.0304	-83.4604
Trailing	Precip	RG96	894	35.0454	-83.4541
Trailing	Precip	RG13	961	35.0624	-83.4562
Trailing	Precip	RG12	1001	35.0473	-83.4586
Trailing	Precip	RG55	1035	35.0399	-83.4554
Trailing	Precip	RG05	1144	35.0605	-83.4650
Trailing	Precip	RG31	1366	35.0327	-83.4681
Core	Temp	HQ	261	43.9457	-71.7010
Core	Temp	STA1	478	43.9521	-71.7248
Core	Temp	STA23	666	43.9272	-71.7469
Core	Temp	STA6	728	43.9572	-71.7349
Core	Temp	STA14	732	43.9208	-71.7656
Core	Temp	STA17	903	43.9190	-71.7582
Core	Precip	RG22	261	43.9440	-71.7012
Core	Precip	RG1	490	43.9521	-71.7248

Core	Precip	RG2	560	43.9550	-71.7270
Core	Precip	RG4	565	43.9552	-71.7208
Core	Precip	RG19	595	43.9293	-71.7594
Core	Precip	RG23	665	43.9272	-71.7469
Core	Precip	RG14	730	43.9208	-71.7656
Core	Precip	RG6	745	43.9572	-71.7349
Core	Precip	RG9	760	43.9557	-71.7418
Core	Precip	RG17	895	43.9190	-71.7582

Notes. Climate stations recorded measurements of either average daily temperature (Temp) or total daily precipitation (Precip). The USDA Forest Service ID (Station ID), elevation, and geographic coordinates (Latitude/Longitude) of climate stations are shown.

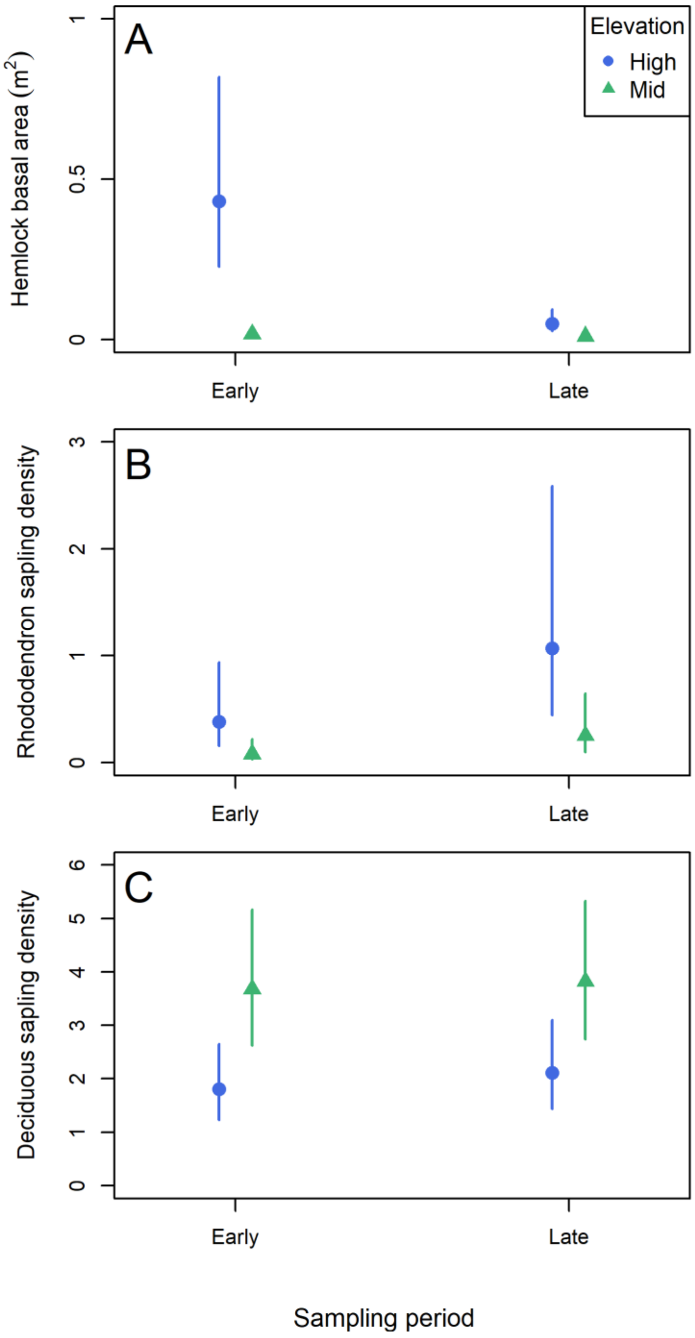

Figure S1. Change in vegetation composition between 2002/2003 (Early) and 2019/2020 (Late) at the trailing edge of the black-throated blue warbler (*Setophaga caerulescens*) breeding range (Appalachian Mountains of North Carolina). Change in average hemlock basal area (A), stem density of sapling *Rhododendron maximum* (the preferred warbler nesting substrate at the trailing edge) (B), and stem density of deciduous saplings (C) at vegetation survey locations is shown for

the mid-elevation (green triangles) and high-elevation (blue circles) study plots. Vegetation was surveyed in both sampling periods at 36 locations at the mid-elevation plot and 32 locations at the high-elevation plot. Diameter at breast height (DBH) was measured for each tree (DBH>9.9cm) within 11.3m of location center. DBH was converted to basal area. Sapling density was measured by counting every stem (>1.4m tall and 10>DBH>2.5cm) within 3m of location center. Average values of vegetation measures for each study plot and sampling period were estimated with a linear mixed-effects model, incorporating location ID as a random effect. Mean estimates and 95% confidence intervals are shown.
